# The Evolution of Reverse Total Shoulder Arthroplasty—From the First Steps to Novel Implant Designs and Surgical Techniques

**DOI:** 10.3390/jcm11061512

**Published:** 2022-03-10

**Authors:** Julia K. Frank, Paul Siegert, Fabian Plachel, Philipp R. Heuberer, Stephanie Huber, Jakob E. Schanda

**Affiliations:** 1 Vienna Shoulder and Sports Clinic, Baumgasse 20A, 1030 Vienna, Austria; 2 Ludwig Boltzmann Institute for Traumatology, The Research Center in Cooperation with the AUVA, Donaueschingenstraße 13, 1200 Vienna, Austria; jakob.schanda@auva.at; 3 1st Orthopaedic Department, Orthopaedic Hospital Vienna-Speising, Speisinger Straße 109, 1130 Vienna, Austria; paul.siegert@oss.at; 4 Michael Ogon Laboratory for Orthopaedic Research, Orthopaedic Hospital Vienna-Speising, Speisinger Straße 109, 1130 Vienna, Austria; 5 Center for Musculoskeletal Surgery, Campus Mitte, Charité Universitaetsmedizin Berlin, Charitéplatz 1, 10117 Berlin, Germany; fabian.plachel@charite.de; 6 Department of Orthopaedics and Traumatology, Paracelsus Medical University Salzburg, Müllner Hauptstraße 48, 5020 Salzburg, Austria; 7 HealthPi Medical Center, Wollzeile 1/3, 1010 Vienna, Austria; philipp@heuberer.at; 8 Austrian Research Group for Regenerative and Orthopaedic Medicine, 1010 Vienna, Austria; 9 Ludwig Boltzmann Institute of Osteology at Hanusch Hospital of OEGK and AUVA Trauma Center Vienna-Meidling, 1140 Vienna, Austria; stephanie.huber@osteologie.lbg.ac.at; 10 AUVA Trauma Center Vienna-Meidling, Kundratstraße 37, 1120 Vienna, Austria; 11 Austrian Cluster for Tissue Regeneration, 1200 Vienna, Austria

**Keywords:** reverse total shoulder arthroplasty, biomechanics, implant design, patient-specific instruments, computer navigation

## Abstract

Purpose of Review: The purpose of this review is to summarize recent literature regarding the latest design modifications and biomechanical evolutions of reverse total shoulder arthroplasty and their impact on postoperative outcomes. Recent findings: Over the past decade, worldwide implantation rates of reverse total shoulder arthroplasty have drastically increased for various shoulder pathologies. While Paul Grammont’s design principles first published in 1985 for reverse total shoulder arthroplasty remained unchanged, several adjustments were made to address postoperative clinical and biomechanical challenges such as implant glenoid loosening, scapular notching, or limited range of motion in order to maximize functional outcomes and increase the longevity of reverse total shoulder arthroplasty. However, the adequate and stable fixation of prosthetic components can be challenging, especially in massive osteoarthritis with concomitant bone loss. To overcome such issues, surgical navigation and patient-specific instruments may be a viable tool to improve accurate prosthetic component positioning. Nevertheless, larger clinical series on the accuracy and possible complications of this novel technique are still missing.

## 1. Introduction

The idea of reverse total shoulder arthroplasty (RTSA) was first introduced in 1974 by Charles Neer and has considerably progressed ever since [1,2,3]. The great novelty of RTSA was its ability to treat not only glenohumeral arthrosis, but also rotator cuff deficiency. Nevertheless, Charles Neer’s first design had several limitations such as glenoid component loosening and implant breakage due to fairly constrained designs as well as a lateralization of the center of rotation [3,4,5].

In 1985, Paul Grammont introduced the novel “ball-and-socket” design, which was based on four key principles: (1) shifting the center of rotation medially to decrease the mechanical torque at the glenoid component, thus avoiding glenoid loosening; (2) lowering the humerus to tension the deltoid muscle, which increases muscle fiber recruitment of the anterior and posterior deltoid in order to compensate a deficient rotator cuff; (3) a fixed center of rotation distalized and medialized to the glenoid joint line, leading to an inherently stable implant; (4) a large glenosphere increasing the range of motion through a semi-constrained implant feature [1,6]. In 1987, the first series of eight cases reported preliminary functional outcomes: all patients were pain-free, however the postoperative range of motion varied widely among patients [2,7]. Unsatisfied with these results, the second generation of Paul Grammont’s RTSA had a revised glenosphere with the shape of a hemisphere to place the center of rotation directly in contact with the glenoid surface in order to reduce shear forces and mechanical torque on the implants and ensure a strong fixation [2,3]. Over time, several changes in implant designs were performed in order to reduce complications and expand the surgical indications of RTSA from the rotator cuff-deficient osteoarthritis [8] to proximal humerus fractures [8], irreparable rotator cuff tears without glenohumeral osteoarthritis [9], primary glenohumeral osteoarthritis with concomitant glenoid bone loss and an intact rotator cuff, and even infections or patients with a deltoid palsy.

**Figure 1:**
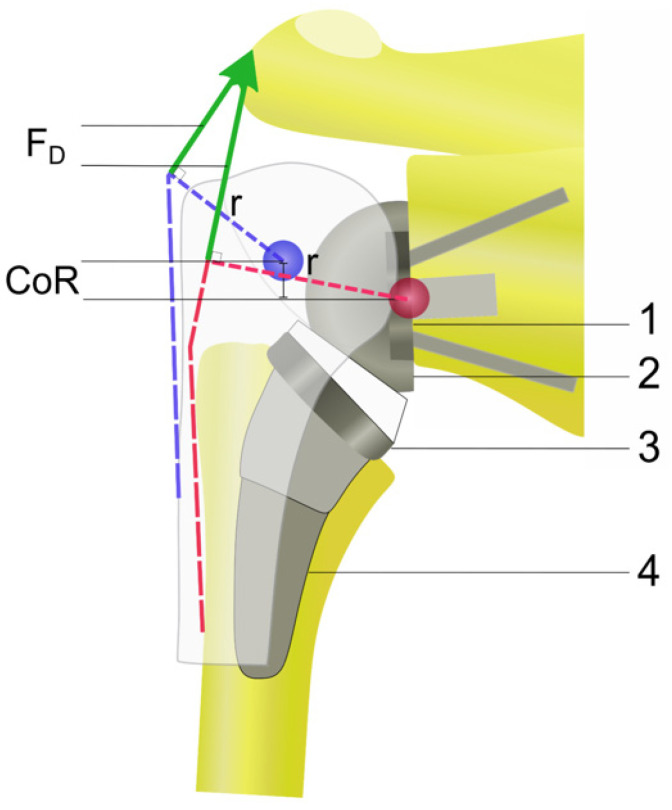
Illustration of biomechanical properties in native joint and reverse total shoulder arthroplasty. Relative to the normal anatomy, the center of rotation (CoR) is shifted medially and inferiorly, thus lengthening the moment arm (r) and increasing the deltoid force (F_D_). 1: glenoid baseplate; 2: glenosphere; 3: metaphyseal component; 4: humeral stem.

Although RTSA has been performed for more than 30 years, varying outcomes have been published, reporting complication rates ranging from 0 to 69% [2,10,11,12,13,14,15,16]. Therefore, research continues tirelessly on the development of new implant designs to maximize function and extend implant survival [17]. Even if the prosthetic properties of different implants have variable biomechanical and kinematic implications, the principles of RTSA consisting of the glenoid baseplate with diverging polar screws and two equatorial screws, the glenosphere shaped as a hemisphere, the humeral stem(-less) component including a modular metaphyseal implant, and the articulating polyethylene inlay remain unchanged (Figure 1).

## 2. Biomechanical Considerations

**Figure 2:**
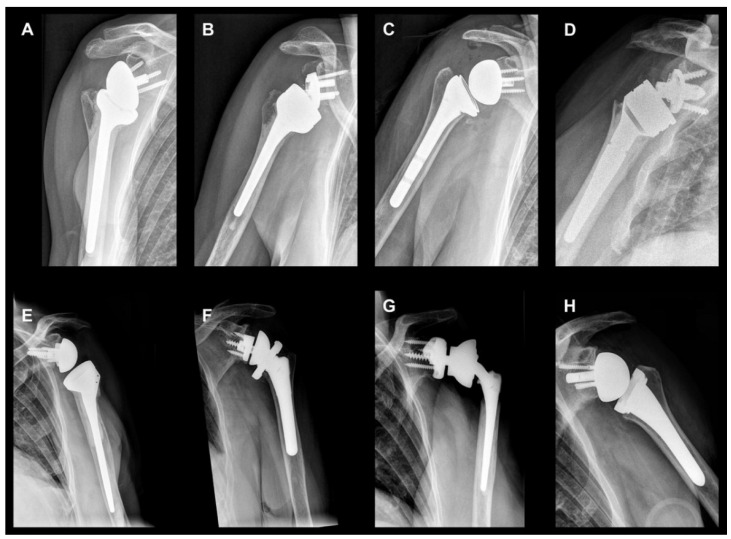
Standard anteroposterior radiographs of the shoulder after reverse total shoulder implantation with different designs and implantation techniques: (**A**) Right shoulder, cemented Delta XTEND™ (DePuy Synthes, Raynham, MA, USA); (**B**) Right shoulder, cemented Affinis Inverse (Mathys, Bettlach, Switzerland); (**C**) Right shoulder, uncemented HUMELOCK™ Reverse (FX Solutions, Viriat, France); (**D**) Right shoulder, cemented Lima SMR-system (Lima, Villanova, San Daniele del Friuli, Italy); (**E**) Left shoulder, cemented Tornier AEQUALIS™ ADJUSTABLE REVERSED (Wright, Memphis, TN, USA); (**F**) Left shoulder, uncemented Comprehensive^®^ Reverse Shoulder System (Zimmer Biomet, Warsaw, IN, USA); (**G**) Left shoulder, cemented custom made reverse total shoulder arthroplasty (Zimmer Biomet, Warsaw, IN, USA); (**H**) Left shoulder, uncemented MyShoulder^®^ (Medacta, Castel San Pietro, Switzerland).

From a biomechanical point of view, modern modifications of implant designs and component’ configurations are still based on the four key principles of Paul Grammont’s design (Figure 2). Compared to the anatomic shoulder replacement, the center of rotation in RTSA is distalized and medialized in order to be parallel to the glenoid joint line [3]. Since all joint reactive forces are transmitted through a fixed center of rotation, the design of the RTSA maximizes compressive forces while minimizing shear forces at the bone implant interface [18]. The hemisphere shape of the glenoid component leads to a direct reduction in the center of rotation to the bone implant interface [6,19]. However, the medialization of the center of rotation carries the risk of scapular notching in the humeral component with the attached inlay. This mechanical impingement of the humeral component and the inferior scapular neck during adduction of the arm is still considered to be the primary mechanical complication in RTSA [19,20].

By distalization and medialization of the center of rotation, RTSA changes the biomechanics of the glenohumeral joint in a way that maximizes deltoid efficiency, thus enabling the patient’s deltoid muscle force by permission of a higher preload of the muscle fibers and a reduction in the deltoid muscle in relation to the joint’s center of rotation [2,21]. This leads to unavoidable and severe changes in the deltoid muscle as fibers are oriented more vertically and all three sub-regions of the deltoid muscle become primary shoulder abductors (Figure 1) [3,22,23]. Several studies investigating the postoperative range of motion reported that deltoid abduction effectiveness increases up to 30% compared to the native anatomy [21,24,25]. In RTSA, the deltoid’s abduction moment arm has much greater fluctuation, peaking at 90° of abduction, the position at which the weight of the arm creates its greatest adducting moment [3,18,26]. Hamilton et al. stated that, especially in patients with rotator cuff tear arthropathy, only the posterior deltoid is left to assist with external rotation [27].

## 3. Implant Design

### 3.1. Glenoid Baseplate

After the failure of a directly cemented fixation of the two-thirds of a sphere-shaped glenosphere to the glenoid bone stock, the original design of the glenoid baseplate, according to Paul Grammont, was fixed using a central press-fit, hydroxy-apatite-coated cylindric peg and two additional peripheral divergent screws [7]. Nowadays, several designs of the glenoid baseplate with different fixation possibilities are available.

Anatomically, a pear-shaped or ovoid glenoid baseplate may improve primary implant fixation and bone ingrowth by an increased contact area of the implant to the glenoid [17]. In a biomechanical study, Roche et al. reported of a reduced fixation strength of a circular glenoid baseplate compared to a larger oval baseplate [28]. Theoretically, a convex-curved glenoid baseplate following the glenoid’s anatomical concavity allows a larger contact area between the implant and the bone [29]. However, Roche et al. reported no differences in fixation strength when comparing a flat glenoid baseplate with a convex-curved glenoid baseplate [28].

Regarding different anchoring possibilities for glenoid baseplate fixation, no scientific evidence on superior or inferior stability exists between screwed, keeled, single-pegged, or double-pegged glenoids [30,31]. However, in the case of severe glenoid bone loss or an additional glenoid baseplate augmentation, the central glenoid baseplate fixation needs to be extended in order to ensure a sufficient primary fixation strength [17]. Norris et al. reported on a lengthened central peg of 30 mm in order to gain a purchase of over 10 mm in the native glenoid bone [32].

For the ultimate fixation of the glenoid baseplate, cortical non-locking screws with compression, locking screws, and compression screws can be used [17]. However, no differences in implant stability were reported after comparing these different screw types [33]. Moreover, screw divergence has no impact on implant stability when compared to parallel screw fixation [34]. Nevertheless, screw length correlates with improved glenoid baseplate fixation, and a higher fixation strength can be achieved in the case of the implantation of four screws compared to only two screws [35].

To overcome complications of glenoid baseplate loosening in the early designs of RTSA, the center of rotation was consequently medialized to reduce the mechanical torque at the glenoid component [2,36]. As a result, higher rates of scapular notching were observed [36]. Reviewing the literature, incidences of scapular notching following RTSA ranged from 4.6% to 96% [37,38,39,40]. A mechanical impingement between the humeral component and the inferior scapular neck and the glenoid were discussed to be responsible for scapular notching [3,6,41]. This mechanical impingement can lead to wear of the polyethylene insert of the humeral component and subsequently cause osteolysis of the surrounding bone [3,42]. However, the clinical significance of scapular notching is still a controversial topic. Several studies have suggested a negative effect on clinical outcomes or an increasing instability of the glenoid component [37,39,43]. In a recently published large outcome study, Mollon et al. demonstrated that scapular notching does have a significant negative influence on clinical outcomes, showing higher complication rates compared to patients without diagnosed scapular notching [40].

To overcome this issue, a lateralization of the glenoid side relative to the medialized center of rotation was expected to reduce postoperative scapular notching [43]. In a biomechanical study, Gutiérrez et al. reported that the largest effect on both abduction and adduction is related to a lateralization of the center of rotation [44]. In a multicenter study analyzing the use of a lateralized glenosphere, improvements in external rotation paired with relatively low rates of scapular notching of 13.3% as well as acromial stress fractures of 6.5% were reported [45]. However, this might again cause excessive motion, leading to glenoid baseplate loosening, the migration of the glenosphere, and ultimately the failure of RTSA [2,12].

**Figure 3:**
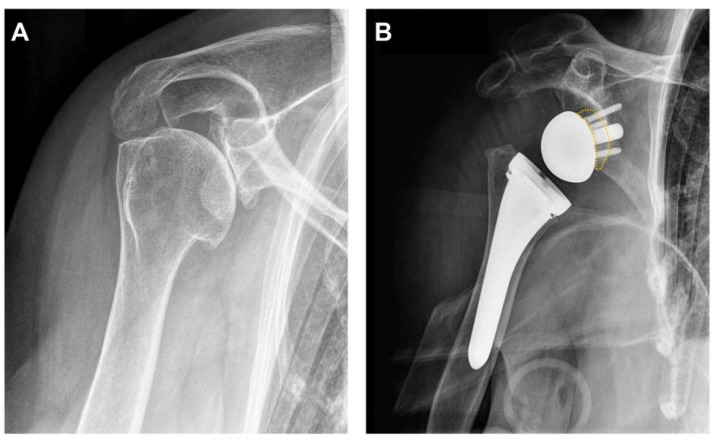
Standard anteroposterior radiographs of a right shoulder before and after implantation of a reverse total shoulder arthroplasty: (**A**) Cuff arthropathy of the shoulder with increased posterior glenoid wear. (**B**) Implantation of an uncemented MyShoulder (R) (Medacta, Castel San Pietro, Switzerland). Additionally, a bony lateral increased-offset harvested from the humeral head was implanted between the glenoid baseplate and the native glenoid (yellow dotted line).

Lateralization of the glenosphere by using a bony augmentation or a metal-augmented baseplate represents a novel option to overcome complications such as scapular notching and compromised external rotation [46,47]. The choice between an additional bone graft and/or a metal-augmented baseplate depends on the implant design as well as the amount of the remaining glenoid bone stock and concomitant wear patterns [48,49,50]. In 2011, Boileau et al. first reported of a “bony increased-offset reverse shoulder arthroplasty” (BIO-RSA), whereby lateralization was achieved by placing a 1 cm thick autologous bone graft harvested from the resected humeral head between a specially designed baseplate and the native glenoid (Figure 3) [51]. Until now, there has only been little evidence on the use of BIO-RSA [51,52]. Of 42 patients, complete bony graft incorporation within the anatomical glenoid bone stock was observed in 98%. Clinical results showed a gain in active mobility, in active anterior elevation as well as in active external rotation. In 86%, the patient’s internal rotation was sufficient enough to reach the back over the sacrum. Furthermore, scapular notching rates were reduced using a BIO-RSA augmentation [51]. A recent study, comparing standard RTSA without lateralization of the glenoid baseplate and the use of a BIO-RSA augmentation, reported a significantly greater active external rotation and a concomitant decrease in scapular notching by using a bony glenoid augmentation [53]. On the other hand, Collin et al. found no clinical of radiological differences comparing bony-augmented glenoid baseplates with standardized glenoid baseplate implantation without lateralization of the center of rotation [54]. Similar to the BIO-RSA, Katz et al. proposed a lateralization of the center of rotation using a symmetric thick baseplate [55]. Lateralization using metal-augmented glenoid baseplates provides improvements in abduction, adduction, as well as in internal and external rotation with a concomitant reduction in scapular notching [44]. Werner et al. reported a significant improvement in active internal rotation using different thicknesses of glenoid baseplate components lateralizing from 6 mm to 8 mm [56].

Recent studies have pointed out the importance of the superior–inferior orientation of glenoid erosion. Humeral head migration in rotator cuff arthropathy might influence the pattern of glenoid wear [57,58]. In these cases, full-wedged or half-wedged augmented baseplates can be useful to compensate for severe glenoid bone loss [17]. Jones et al. compared autologous glenoid bone grafting with the use of metal-augmented baseplates, suggesting that both designs showed favorable results in addressing large defects and improved clinical outcomes. However, metal-augmented baseplates may result in lower rates of scapular notching with similarly lower complications such as graft resorption and glenoid baseplate loosening [59,60,61,62]. Recently, Nabergoj et al. reported of satisfying clinical and radiological outcomes in patients with RTSA and concomitant bony and metallic augmentation for severe glenoid bone loss compared to patients with bony glenoid augmentation only [63].

### 3.2. Glenosphere

The original glenosphere, according to Paul Grammont, was designed corresponding to two-thirds of a sphere and thereby led to a medialization of the center of rotation [7]. Glenosphere design and implantation were constantly adapted regarding diameter, inferior, and lateral offset, as well as inclination, in order to reduce complications such as scapular notching or impaired range of motion [17].

To decrease polyethylene wear and concomitant osteolysis, several studies focused on the inversion of mechanical bearings in RTSA, resulting in a glenosphere made of polyethylene and an articulating humeral liner consisting of metal or ceramic [64,65,66]. Preclinical data showed significantly less polyethylene bearing in the case of inverted bearing materials for RTSA [64,65]. First clinical studies confirmed the safety of polyethylene glenospheres without adverse effects such as polyethylene-induced osteolysis [65,66]. However, these novel polyethylene glenospheres with the articulating metallic humeral liner presented increased rates of scapular notching with pathognomonic sclerotic lines at the lateral glenoid border, caused by the mechanical impingement of the metallic humeral liner [64,66].

To improve the range of motion after RTSA, larger diameter glenospheres became more popular over the last years. In a cadaver study, a glenosphere with a diameter of 42 mm and a lateralized spacer of 7 mm or 10 mm showed the best range of motion for internal and external rotation. However, active range of motion is dependent on muscle strength and can therefore not be evaluated in a cadaver study [67]. Mollon et al. showed that a glenosphere with a larger diameter of 42 mm significantly improved active forward elevation and external rotation compared with a glenosphere with smaller diameter of 38 mm [68]. Even a larger glenosphere with a diameter up to 44 mm lead to a significant increase in external rotation as well as an improved abduction and adduction strength [69]. In the case of revision surgery due to recurrent prosthetic joint dislocation, the implantation of a larger glenosphere can increase deltoid tension and thereby achieve a higher joint stability [70,71]. Even if a glenosphere with a larger diameter increases joint stability and the postoperative range of motion, the clinical implications remain unclear, as a larger glenosphere may not be feasible for the anatomy of smaller patients [72,73,74].

Lateralization of 6 mm to 8 mm was shown to positively improve active axial glenohumeral rotation and impingement-free range of motion [44,53,56,75]. On the other hand, scapular notching can be reduced by shifting the cranio-caudal position of the glenosphere. As an advantage, the range of motion can be improved by impeding the lateral offset [3,76]. Moreover, an inferior glenosphere overhang beyond the inferior glenoid border through the eccentric placement of the glenosphere can lead to a greater movement in both adduction and abduction [73,77]. De Wilde et al. evaluated the effect of an inferior glenosphere overhang, showing a reduction in scapular notching with a concomitant increase in adduction [73,78]. Until now, the ideal amount of glenoid tilt remains unclear. However, several studies have suggested that slight inferior tilt may be an advantage in providing a greater impingement-free glenohumeral range of motion [44,74,79]. Negatively, inferior eccentricity of the glenosphere can lead to an excessive distalization of the humerus, causing stretching of the brachial plexus and especially the axillary nerve, with irreversible damage and compromising the deltoid muscle function [55,80].

Besides an inferior overhang, the glenosphere can be implanted with an inferior tilt, leading to further distalization and medialization of the center of rotation with a possible benefit for patients suffering from a rotator cuff tear arthropathy [81]. In a virtually supported cadaver study, Li et al. reported the ideal glenosphere positioning in an inferior translation, inferior tilt, and lateralization in all degrees of scaption [82]. Werner et al. reported an ideal inferior tilt of 10° to be optimal concerning glenohumeral extension and external rotation [75]. As a consequence, novel glenosphere designs are customized with a built-in inferior inclination of 10°, which facilitates implantation with an inferior tilt without risking excessive reaming of the inferior glenoid and thereby compromising implant stability.

Until now, positioning of the glenoid component remains a crucial step in RTSA, since it affects the long-term survival and functional outcomes of prosthesis [41,83]. Inaccurate positioning of the glenoid can lead to instability, loosening, and ultimately failure of RTSA [84,85].

### 3.3. Humeral Component

In his first design, Paul Grammont proposed a long and straight cemented stem [7]. However, due to radiographic changes surrounding the stem, with high rates of bone resorption around the tuberosities eventually leading to early humeral component loosening and impaired range of motion because of increased stress shielding, new designs of uncemented and proximally coated humeral components for press-fit fixation were developed [86,87,88].

An important point in humeral component implantation was the decision on the use of bone cement to ensure a sufficient fixation. In recent years, uncemented humeral fixation has become more successful and has allowed the development of bone preserving short-stem and stemless humeral prosthesis [89,90,91]. Novel curved-type humeral components with a proximal hydroxyapatite-coated interface have only recently become more popular. The shortening of the humeral component leads to a rather physiological distribution of shear forces and mechanical torque [92]. Thereby, a greater amount of bone can be preserved, and the occurrence of tuberosity fractures is less likely [6,93]. To preserve even higher amounts of humeral bone stock and to reduce metaphyseal stress shielding, stemless designs for RTSA were introduced in 2004 with either an impaction or a screw fixation. Stemless prostheses depend on proximal bone stock and rely on subscapularis repair. Various studies describe the positive effects of stemless implants including bone preservation, decreased blood loss, less distal stress shielding, and less lateralization [90,91,94,95]. Moroder et al. reported short to mid-term follow-up rates following stemless RTSA in a case-control study [89]. After a mean follow up of 35-month stemless RTSA, 37 patients were compared to standard RTSA. The authors observed satisfactory radiological and clinical outcomes including pain relief and patient satisfaction in the stemless RTSA group. [89]. Levy et al. evaluated the clinical and radiologic outcomes at two to seven years using a short metaphyseal RTSA, showing satisfying short- to mid-term results with great pain relief and the restoration of a good active range of motion [96]. However, there is only a limited number of trials for stemless implants and long-term survivorships still need to be evaluated.

Limitations in internal and external rotation remain a challenge in modern RTSA [16]. Rotational movement in the shoulder results from a spin around its center. While the native joint has its center in the midpoint of the humeral head, in RTSA, the humeral component rotates around the glenosphere due to the medialization of the fulcrum [2,97]. Biomechanical investigations showed that the rotational movement is influenced by the torsion placement of the humeral component [2,97,98]. While internal rotation is increased by a lower retrotorsion, external rotation is facilitated by a higher retrotorsion [98]. Until now, the ideal humeral component torsion is still a matter of debate [2,3,97,98,99]. While Stephenson et al. proposed an overall advantage in range of motion with a retrotorsion of 20° to 40° [98], Favre et al. recommended an implantation at neutral rotation or even anteversion in order to improve intrinsic stability [100]. A recent study by Moroder et al. proposed the integration of patient posture for individualized retrotorsion angles [101]. The authors concluded that, in the case of an advanced kyphosis, as well as a protracted and internally rotated scapula, the use of an increased angle of humeral component torsion should be considered [101].

### 3.4. Polyethylene Humeral Inlay

The original polyethylene humeral inlay from Paul Grammont was designed at a neck-shaft-angle (NSA) of 155° with a depth of 8 mm [7]. Since then, several changes and adaptation have been performed on the polyethylene cup. The contact area between the glenosphere and the polyethylene inlay is one of the key factors for stability in RTSA. Several inlays ranging from high-mobility to constrained or retentive versions are available [44,73,102].

The NSA plays a crucial role in implant stability and has a major influence on clinical outcomes. The NSA is measured by a line along the central intramedullary axis of the humeral shaft and a tangent passing through the anatomic neck-head junction [103,104]. According to Gutiérrez et al., the NSA has a major impact on inferior scapular notching and adduction deficiencies. A smaller NSA resulting in a varus positioning significantly improves the adduction capacity [44]. Although the risk of scapular notching can be reduced using a polyethylene inlay with a smaller NSA, a major improvement was observed by using a shorter humeral stem with a varying NSA. Lädermann et al. reported a greater range of motion in adduction, extension, and external rotation by using a shorter and curved stem with different NSAs ranging between 135° and 155°, especially in case of varus NSAs [93]. A systematic review including 38 trials with 2222 shoulders found significantly higher rates of scapular notching in RTSA, with an NSA of 155° compared to an NSA of 135°. However, upon comparing the risk of prosthetic joint dislocation, no differences were observed comparing different NSAs [105].

In the case of prosthetic joint instability, the use of a more constrained or retentive inlay is suggested [106,107]. While retentive inlays contribute to a higher stability, several studies have showed that the risk of increased polyethylene wear and aseptic loosening is increased due to higher shear forces on the articulating partners [106,107]. Moreover, Mueller et al. reported a significantly greater incidence of polyethylene wear and rim damage of the humeral components in the case of scapular notching [108]. By using a two-dimensional model of the scapula showing a change in cup depth from 8 mm to 5 mm, range of motion increased by 12° in abduction [73]. Similarly, Elwell et al. reported of a significant, however probably clinically irrelevant, increase in the overall range of motion by reducing the polyethylene cup depth from 8.1 mm to 6 mm [109]. Lädermann et al. suggested that a polyethylene with a notch between 3 and 9 o’clock may have a positive effect on postoperative range of motion as well as a reduction in scapular notching [110]. Even if prosthetic joint stabilization can be achieved by increasing the thickness of the inlay, overstuffing the articulation may produce unfavorable effects on the deltoid muscle and joint loading [111].

In order to implant the polyethylene cup within the metaphyseal component of the humeral shaft, reaming of the metaphyseal area is mandatory. However, this might cause excessive bone loss, ultimately leading to resorption of the tuberosities. To overcome this issue, another design change was made to RTSA, leading to novel onlay polyethylene liners being placed just above the metaphysis at the level of the humeral head osteotomy. This onlay design results in a lateralization of the humerus by mechanical shifting of the humeral stem from the glenosphere, which might lead to an overstuffing of the joint, eventually causing impaired range of motion or failure of RTSA due to excessive shear forces and mechanical torque [17,112,113]. On the other hand, there are several advantages to an onlay design such as a larger deltoid moment arm for improved deltoid efficiency and better tensioning of the rotator cuff muscles [17,112,114].

## 4. Computer Navigation and Patient-Specific Instrumentation

Since adequate and stable fixation of prosthetic components can be challenging, especially in massive osteoarthritis and concomitant bone loss, surgical navigation and patient-specific instrument (PSI) techniques were first introduced in the mid-to-late 2000s and later adapted in 2014 by Ianotti et al. [115]. PSI and computer navigation were developed to improve glenoid positioning, especially in technically challenging cases with complex and variable anatomy [116,117]. Sadoghi et al. performed a meta-analysis assessing data for glenoid version for 117 navigated shoulder replacements and 114 standard prostheses. In navigated shoulders, the error for implant version and inclination was significantly reduced [118]. Over the past years, technology has progressed, and patient-specific guides based on three-dimensional printed models of the patient’s anatomy and the matching cutting blocks are becoming gradually commercially available. The accuracy of preoperative planning facilitates the prediction of the correct implants and may optimize supply chain logistics for both hospitals and implant companies [119].

Several software tools are available using standard preoperative computed tomography scans of the shoulder to define the optimal positioning of the implants [116,120]. Current software use bony anatomy to estimate the postoperative range of motion and predict early impingement. However, scapulothoracic orientation has not yet been considered. In a recent study, Moroder et al. [121] investigated the influence of patients’ posture on the range of motion following RTSA implantation in a modelling study. They used a modified preoperative planning software with a coordinate system to include scapular orientation in relation to the thorax.

Ultimately, patient-specific drill guides became available for the surgical procedure [122]. Until now, only controversial data on the use of PSI has been published. Lau et al. suggested that the accuracy of PSI-guided glenoid position is not as favorable [123]. On the other hand, Dallalana et al. reported PSI to be accurate and highly reliable in optimal orientation of the glenoid [124]. A recently performed meta-analysis reported that computer navigation and PSI lead to better glenoid positioning outcomes. However, due to the heterogeneity of results, it remains unclear whether these improvements are beneficial for patients’ clinical outcome [125].

One of the most common failures following RTSA is the malpositioning of the glenoid component, which is associated with increased humeral instability, leading to inferior outcomes and ultimately to component loosening [36,83,126].

A new approach to improve RTSA is the use of mixed reality-guided implantation. Augmented reality, a technique that allows the superimposing of a digital image on top of the visual field, may be a key tool for the future [116]. In 2018, Gregory et al. first described the technology of a mixed reality headset enabling the hand-user the interaction by oral command or simple gesture. During the surgery, data of the operative technique can be transmitted into the operating headset in real time without prolonging the surgical time [127]. However, larger clinical series on accuracy and possible complications of this novel technique are still missing.

Despite satisfying clinical results [124,125], the use of PSI still faces challenges such as logistics due to preoperative scanning of the shoulder and manufacturing of custom-made cutting blocks and implants paired with higher costs compared to conventional RTSA.

## 5. Conclusions

RTSA has revolutionized the management of many shoulder pathologies over the past years. Constant changes in prosthetic designs were made to overcome complications such as implant loosening, scapular notching, or limited range of motion. By the medialization of the glenohumeral center of rotation, as originally suggested by Paul Grammont in his first design of RTSA, deltoid abduction effectiveness could be increased by up to 30% compared to native anatomy. On the other hand, lateralization of the center of rotation using an autologous bone graft or a metal-augmented baseplate is associated with lower rates of scapular notching and improved shoulder rotation capacity. The use of novel PSI techniques and computer navigation may be a viable tool with which to improve accurate component positioning, leading to a patient-oriented, tailor-made RTSA.
